# Mortality among mine and mill workers exposed to respirable crystalline silica

**DOI:** 10.1371/journal.pone.0274103

**Published:** 2022-10-14

**Authors:** Sarah E. Kleinschmidt, Kara L. Andres, Brian M. Holen, Betsy D. Buehrer, Gerardo Durand, Oyebode Taiwo, Geary W. Olsen

**Affiliations:** 1 Corporate Occupational Medicine Department, 3M Company, St. Paul, MN, United States of America; 2 Industrial Mineral Products Division, 3M Company, St. Paul, MN, United States of America; Ruhr University Bochum, GERMANY

## Abstract

**Background:**

Millions of workers are potentially exposed to respirable crystalline silica (RCS) which has been associated with several diseases. We updated the mortality experience of a cohort of 2,650 mine and mill workers at four manufacturing facilities to assess cause-specific mortality risks associated with estimated cumulative RCS exposure.

**Methods:**

Study eligibility was defined as any employee who had ≥1 year of service by 2000, with work history experience available from 1945 through 2004. Vital status and cause of death were ascertained from 1945 through 2015. RCS exposure was estimated across plant-, department-, job-, and time-dependent categories using historic industrial hygiene sampling data and professional judgment. Associations between cumulative RCS (mg/m^3^-years) and cause-specific mortality were examined using Cox proportional hazard regression models.

**Results:**

In the exposure-response analysis defined on quartiles of cumulative RCS exposure, no increasing trend (p_trend_ = 0.37) in lung cancer mortality (n = 116 deaths) was observed (Hazard ratio (HR) = 1.00 (referent), 1.20, 1.85, 0.92). Mortality risk for non-malignant respiratory disease was increased across quartiles (HR = 1.00, 1.35, 1.89, 1.70; p_trend_ = 0.15), based on 83 deaths. Non-malignant renal disease mortality was increased across quartiles (HR = 1.00, 6.64, 3.79, 3.29; p_trend_ = 0.11), based on 26 deaths.

**Conclusions:**

After nearly seven decades of follow-up, the exposure-response analyses showed no evidence of a positive trend for lung cancer, and limited evidence of a trend for non-malignant respiratory disease, and non-malignant renal disease mortality as a result of cumulative RCS exposure in this occupational cohort.

## Introduction

The Occupational Safety and Health Administration (OSHA) estimates that more than 2.3 million U.S. workers are potentially exposed to crystalline silica in the workplace [1]. These include more than 100,000 workers in high-risk jobs, such as abrasive blasting, foundry work, stonecutting, rock drilling, and quarry work. In 2016, OSHA lowered the permissible exposure limit for crystalline silica to 0.05 mg/m^3^.

Occupational exposure to respirable crystalline silica (RCS) has been associated with several diseases [2]. Silicosis, a progressive and often fatal fibrotic lung disease, is caused by inhaling RCS. Silicosis is characterized by chronic inflammation and scarring in the form of nodular lesions especially in the upper lobes of the lungs. Silicosis mortality has declined substantially over the past several decades, which is largely attributed to lower occupational exposure limits and improved preventive measures of exposure.

Workers exposed to RCS may also be at increased risk for non-malignant respiratory diseases other than silicosis. Epidemiologic evidence suggests that chronic silica exposure may contribute to the development of bronchitis, emphysema, and/or small airway disease, which can lead to chronic obstructive pulmonary disease (COPD) [3]. However, the risk of non-malignant respiratory disease is influenced by smoking, and the effects of silica exposure and smoking may be synergistic [4].

Since 1997, the International Agency for Research on Cancer has concluded that crystalline silica, inhaled in the form of quartz or cristobalite from occupational sources, is carcinogenic to humans. The strongest supportive evidence for the carcinogenicity of crystalline silica on the lung comes from pooled and meta-analyses [5–10]. The increased risk of lung cancer observed in humans is also supported by tumor site concordance with carcinogenesis studies in rodents and additional mechanistic support, although it is unclear which mechanism predominated [11].

Several cohort studies of silica-exposed workers have observed an association with non-malignant renal disease incidence and/or mortality [12–16], while other studies have not [17–20]. Given the heterogeneity of results between studies, and no clear evidence of an exposure-response, the actual contribution of RCS exposure on non-malignant renal disease remains unclear.

The objective of this epidemiologic research was to update the mortality experience of a cohort of 2,650 employees in relation to potential workplace exposure to RCS. This study extended mortality follow-up by 11 years from the initial mortality study which observed a significant trend in non-malignant respiratory disease with cumulative RCS exposure, a lack of any well-defined association with lung cancer and an imprecise and nonsignificant association with non-malignant renal disease [21]. The primary *a priori* outcomes of interest for the present analysis were lung cancer, non-malignant respiratory diseases, and non-malignant renal disease, in relation to cumulative RCS.

## Materials and methods

### Study population

The present study utilized the cohort identified in the initial mortality study of 2,650 employees in the mine and mill production of roofing granules which began at four 3M Company manufacturing facilities located in Belle Mead, NJ (starting in 1961); Corona, CA (starting in 1948); Little Rock, AR (starting in 1947); and Wausau, WI (starting in1945), as described previously [21]. The primary manufacturing activity at these four facilities has been open pit quarry mining of rock. The rock is then crushed and screened to produce colored roofing granules for use in asphalt shingles.

Of the four 3M roofing granule manufacturing facilities, the Belle Mead, NJ facility is the newest and smallest. Roofing granule production began at Belle Mead in 1961 and the facility was sold by 3M in 2009. The finished product at this facility had less than 2% crystalline silica by weight and the raw rock contained 2.5% crystalline silica, by weight, along with traces of asbestiform actinolite [22]. Manufacturing began in the Corona, CA plant in 1947 and remains in operation. The raw rock at the Corona facility contained an average of 27.6% crystalline silica, by weight and the finished product was 25–30% crystalline silica, by weight. The Little Rock, AK is the largest of the four manufacturing facilities. It was acquired by 3M in 1947 and remains operational. It had approximately 1.2% crystalline silica, by weight, with the finished product containing less than 1.5% crystalline silica. The plant in Wausau, WI was acquired by 3M in 1929 and began production of roofing granules in 1933 with employment records extending back to 1945 to the present. The raw rock at the Wausau facility contained 21.1% crystalline silica, by weight, while the finished product contained 25–35% crystalline silica [22].

All subjects had their work history followed through December 31, 2004. Because our interest was in the mortality experience of the original study cohort, more newly hired and less potentially RCS exposed employees were not added to the cohort in this update. Original study eligibility was defined as any employee who had ≥ 1 cumulative years of service since the above start dates. For each location, the cumulative 1 year of service had to occur by December 31, 2000. The 3M Institutional Review Board (St. Paul, MN) reviewed and approved this study. Written informed consent was not obtained for this cohort mortality study as this study was based on company records with no individual contact with the workers.

### Vital statistics determination

This cohort was previously studied for cause-specific mortality through 2004 using searches from the Social Security Administration (SSA) (pre-1979) and the National Death Index (NDI) since 1979. Deaths prior to 1979 were identified through the SSA which notifies the requestor that a death has occurred and in which state. However, information regarding the cause of death is not provided. Therefore, copies of death certificates were requested from the state of record in order to have a nosologist code the underlying cause of death information.

For the current update, an NDI Plus search was used to identify additional employee deaths and their underlying causes of death from 1945 through December 31, 2015. All underlying causes of death were coded to ICD9 using an online conversion program (ICD 9 to ICD10 Bidirectional Crosswalk Tool, www.icd10cmcode.com). Employees who were not identified as deceased were presumed to be alive as of the end of follow-up. In the original study, 25 individuals were considered lost to follow-up if they were not found deceased from NDI or Social Security by end of study and had a last work history date before January 1, 1979, but were 50 or more years of age on this date [21]. In this updated study, two of these individuals were found to be deceased; 23 remain lost to follow-up.

### Exposure assessment

Hewitt et al. [22] provides the detailed methods by which RCS exposure through 2004 was determined and the job exposure matrix (JEM) was created. Briefly, historic industrial hygiene sampling data and professional industrial hygiene judgment were used to construct a total of 15 RCS exposure categories from a total of 1,871 sampling measurements. The mean respirable silica (mg/m^3^) values for these 1,871 measurements were: All plants = 0.0956 mg/m^3^ (standard deviation (SD) 0.1922); Belle Mead = 0.0425 mg/m^3^ (SD 0.0599); Corona = 0.1055 mg/m^3^ (SD 0.1432); Little Rock = 0.0507 mg/m^3^ (SD 0.1060); and Wausau = 0.1521 mg/m^3^ (SD 0.2884). An exposure category was assigned to all plant-, department-, and time-dependent standard job titles. These standard job titles were developed from unique plant-, department-, job-, and time-dependent listings that were created from the department codes, job titles, and dates contained in the computerized work history records for the plants. The average of the exposure category was assigned to the time-dependent, department-related, plant-specific standard job titles and this constituted the JEM.

Subsequent to the creation of the standard job titles and the JEM, the appropriate RCS exposure category mean was assigned to each job in the work history for every eligible cohort member. The basic process was performed by plant as follows: 1) for each job in the work history the job title was converted to the corresponding standard job title by matching on department code, work history job title, and time interval; and 2) for that job, the RCS exposure category mean was assigned by matching on department code, standard job title, and time interval. When any one of the key work history variables was missing, or the standard job titles or JEM were incomplete, professional investigator judgment was used to assign the RCS exposure category mean to the given job. Guidelines were created to aid this professional investigator judgment as presented in Hewett et al. [22]. Additionally, unweighted mean RCS exposure values, based on the JEM, were calculated for each plant overall and for each of the individual departments within the plants.

Using the Occupational Cohort Mortality Analysis Program (OCMAP-Plus, University of Pittsburgh, Pittsburgh, PA), cumulative RCS exposure (mg/m^3^-years) through 2004 was calculated for each cohort subject as defined in Eq 1 below.

*Eq1*

$$\mathrm{Individual}\;\mathrm{cumulative}\;\mathrm{RCS}\;\mathrm{exposure}\;(\mathrm{mg}/m^{3}‐\mathrm{years})=\overset{N_{plant}}{\underset{i}{\sum }}\overset{N_{dept}}{\underset{j}{\sum }}\overset{N_{job}}{\underset{k}{\sum }}\overset{N_{year}}{\underset{y}{\sum }}({\bar{C}}_{\mathrm{ijkx}}\;{}^{•}\;t_{\mathrm{ijky}})$$ Eq 1

where the cumulative exposure of each worker was calculated as the product of the average exposure for each plant-department-job-year combination and time spent in that plant-department-job during the yth year, summed across all combinations of plant, department, job, and year. C is the estimated mean for each plant-department-job year combination, and t is the time in months that year for the employee.

### Statistical analysis

Analyses using the cohort as an internal comparison were conducted through Cox proportional hazard models using SAS Version 9.4 (SAS Institute, Cary, NC). Cumulative RCS exposure was categorized as quartiles of the distribution of exposure among all deaths from each outcome. Tests of linear trend were completed by including the median value for each category of cumulative exposure, among those who died, as a continuous variable. The Cox models were adjusted for sex, race, age at the start of follow-up, and calendar year at start of follow-up, where years of follow-up (person-years) was the time variable. To evaluate the validity of these models, proportional hazards assumptions were assessed for all covariates and the functional form was assessed to detect nonlinearity for all continuous covariates. HRs were analyzed for three time periods: 1945–2004 (the original cohort study), 2005–2015 (the time between the original cohort study and follow-up) and 1945–2015 (the entire study period). In a sensitivity analyses, we restricted the analyses to males only to assess potential residual confounding by sex. Both unlagged analyses and analyses where cumulative RCS exposures were lagged for 15 years were conducted. Although RCS exposure data was not updated after 2004, only 16% of the original cohort was still working at that time. Lagging exposure by 15 years addressed this issue to a large extent since the last 15 years of exposure are excluded in the lagged analysis. It is our intention to include more newly hired individuals (≥2000) in the next study update.

## Results

Descriptive statistics for demographic characteristics are presented in **Table 1**. The total cohort consisted of 2,650 employees (93.3% male and 6.7% female) who met the original eligibility criteria of having worked a cumulative 1-year or longer time period. There were 1,187 (45%) reported deaths in the cohort from all causes from 1945 to 2015 (415 additional deaths since the last follow-up, 1945–2004) and a total of 91,516 person-years of follow-up.

**Table 1:** Demographic distribution and vital status, 1945–2015.

Characteristic	Bell Meade (n = 468)	Corona (n = 723)	Little Rock (n = 895)	Wausau (n = 586)	Total (n = 2,650)
Vital Status					
Alive	294 (62.8%)	381 (52.7%)	472 (52.7%)	309 (52.7%)	1,440 (54.3%)
Deceased	173 (37.0%)	334 (46.2)	422 (47.2%)	264 (45.1%)	1,187 (44.8%)
Lost to follow-up	1 (0.2%)	8 (1.1%)	1 (0.1%)	13 (2.2%)	23 (0.9%)
Sex					
Males	441 (94.2%)	682 (94.3%)	826 (92.3%)	545 (93.0%)	2,473 (93.3%)
Females	27 (5.8%)	41 (5.7%)	69 (7.7%)	41 (7.0%)	177 (6.7%)
Year of birth (median year, range)	1947 (1901–1979)	1943 (1885–1980)	1944 (1891–1981)	1942 (1879–1981)	1944 (1879–1981)
Age at hire (median years, range)	26.9 (18.0–59.6)	29.3 (16.5–72.6)	28.9 (16.8–61.9)	26.7 (17.7–68.2)	28.2 (16.5–72.6)
Duration of employment (median years, range)	4.6 (1.0–37.3)	3.8 (1.0–41.5)	10.8 (1.0–43.9)	9.4 (1.0–40.1)	6.3 (1.0–43.9)
Length of follow-up (median years, range)	36.4 (0.1–54.1)	35.8 (0.1–66.4)	35.5 (0.1–67.7)	36.4 (0.1–68.8)	35.9 (0.1–68.8)
Total person-years	15,820.6	24,537.1	30,741.3	21,007.3	91,516.4

The sum of the four individual manufacturing facilities is greater than the total cohort due to 19 subjects being employed in more than one facility.

The distribution of cumulative RCS exposure for the cohort through 2004 has been previously reported [21, 22]. Cumulative RCS (mg/m^3^-years) ranged from 0.003 mg/m^3^-years to 5.31 mg/m^3^-years for the total cohort. The arithmetic and geometric means of cumulative RCS were 0.43 mg/m^3^-years (95% CI 0.41–0.46) and 0.17 mg/m^3^-years (95% CI 0.16–0.18), respectively, and the 90^th^ percentile was 1.10 mg/m^3^-years. Among the four manufacturing facilities, the geometric mean cumulative RCS exposures were highest for Wausau (0.26 mg/m^3^-years, 95% CI: 0.23–0.30) and Corona (0.23 mg/m^3^-years, 95% CI 0.21–0.25) due to the higher silica content of rock in the quarries. The geometric means for Belle Mead and Little Rock were 0.07 mg/m^3^-years (95% CI: 0.06–0.08) and 0.17 mg/m^3^-years (95% CI: 0.15–0.18), respectively.

**Table 2** presents hazard ratios (HRs) for the total cohort for outcomes of *a priori* interest for unlagged and 15-year lagged RCS exposures by follow-up period. In the unlagged analysis, the HR for the third cumulative RCS exposure category (0.224-<0.456 mg/m^3^) was increased relative to referent group (HR = 1.85, 95% CI: 1.09–3.14), however mortality risk was decreased in the highest exposure category, ≥0.456 mg/m^3^, (HR = 0.92, 95% CI: 0.54–1.58) and there was not an increased trend (p_trend_ = 0.37). Moreover, when cumulative RCS exposure was lagged for 15 years, a *decreasing* trend (p_trend_ = 0.01) in lung cancer mortality risk (HRs = 1.00, 0.75, 1.25, 0.49) was observed.

**Table 2:** Hazard Ratios (HRs) for selected causes of death by cumulative RCS exposure (mg/m3 –years) and follow-up period for the total cohort.

Cumulative Exposure (mg/m^3^ –years)	1945–2004			2005–2015			1945–2015		
	Deaths (n)	HR	95% CI	Deaths (n)	HR	95% CI	Deaths (n)	HR	95% CI
Lung cancer									
No lag									
<0.089	17	1.00	referent	12	1.00	referent	29	1.00	referent
0.089-<0.224	18	1.42	0.71–2.82	10	0.90	0.38–2.09	28	1.20	0.70–2.04
0.224-<0.456	22	2.40	1.24–4.66	8	1.21	0.49–3.02	30	1.85	1.09–3.14
≥0.456	21	1.21	0.62–2.38	8	0.62	0.25–1.54	29	0.92	0.54–1.58
p-value for trend		0.92			0.27			0.37	
15-year lag									
<0.089	20	1.00	referent	14	1.00	referent	34	1.00	referent
0.089-<0.224	14	0.81	0.41–1.63	9	0.71	0.30–1.65	23	0.75	0.44–1.28
0.224-<0.456	20	1.43	0.75–2.71	8	1.07	0.44–2.59	28	1.25	0.75–2.11
≥0.456	13	0.56	0.27–1.15	7	0.50	0.20–1.26	20	0.49	0.28–0.86
p-value for trend		0.12			0.18			0.01	
Non-malignant respiratory disease (excluding influenza/pneumonia)									
No lag									
<0.108	12	1.00	referent	8	1.00	referent	20	1.00	referent
0.108-<0.344	9	0.72	0.30–1.75	13	2.28	0.92–5.67	22	1.35	0.72–2.53
0.344-<0.799	16	1.84	0.84–4.02	5	1.44	0.45–4.53	21	1.89	1.00–3.59
≥0.799	13	1.31	0.58–2.96	7	1.98	0.69–5.67	20	1.70	0.89–3.27
p-value for trend		0.28			0.53			0.15	
15-year lag									
<0.108	15	1.00	referent	8	1.00	referent	23	1.00	referent
0.108-<0.344	9	0.55	0.23–1.27	13	2.30	0.92–5.72	22	1.13	0.62–2.07
0.344-<0.799	12	1.06	0.48–2.33	5	1.47	0.47–4.65	17	1.30	0.68–2.50
≥0.799	13	1.03	0.47–2.22	7	2.12	0.74–6.10	20	1.47	0.78–2.76
p-value for trend		0.50			0.44			0.23	
Non-malignant renal disease									
No lag									
<0.247	5	1.00	referent	1	1.00	referent	6	1.00	referent
0.247-<0.382	4	2.93	0.75–11.39	3	32.27	3.20–325.63	7	6.64	2.17–20.27
0.382-<0.714	2	0.91	0.17–4.93	4	37.04	3.35–409.11	6	3.79	1.15–12.46
≥0.714	5	1.87	0.53–6.56	2	12.63	0.94–170.06	7	3.29	1.05–10.35
p-value for trend		0.47			0.22			0.11	
15-year lag									
<0.247	5	1.00	referent	1	1.00	referent	6	1.00	referent
0.247-<0.382	4	2.79	0.72–10.84	3	33.06	3.27–334.41	7	6.38	2.08–19.58
0.382-<0.714	2	0.91	0.17–4.90	4	36.69	3.35–401.72	6	3.79	1.15–12.46
≥0.714	4	1.52	0.40–5.81	2	13.29	0.98–180.81	6	2.81	0.85–9.22
p-value for trend		0.70			0.15			0.24	

Models were adjusted for sex, race, age at start of follow-up, and calendar year at start of follow-up.

Increased HRs for non-malignant respiratory disease (excluding influenza and pneumonia) by exposure quartiles were observed in both unlagged (HR = 1.00 (referent) 1.35, 1.89, 1.70; p_trend_ = 0.15) and 15-year lagged models (HR = 1.00, 1.13, 1.30, 1.47; p_trend_ = 0.23) during the 1945–2015 period, with no evidence of an exposure-response trend.

Mortality risk for non-malignant renal disease for the total cohort was higher in the 1945–2015 follow-up period (HRs = 1.00, 6.64, 3.79, 3.29; p_trend_ = 0.11). Of the total 26 deaths, 14 (54%) deaths were listed as renal failure (acute or not otherwise specified), 11 (42%) were attributed to chronic kidney disease and one (4%) was attributed to chronic glomerulonephritis (S1 Table). HRs for non-malignant renal disease at Corona and Little Rock were higher, than the other facilities, in both the unlagged and 15-year lagged analyses but were based on few deaths in each exposure quartile and were highly imprecise as evident by their wide confidence intervals (S2–S5 Tables).

In a sensitivity analyses, HR estimates for lung cancer, non-malignant respiratory disease and non-malignant renal disease among males only were not substantially different from HR estimates for the entire cohort (S6 Table).

## Discussion

This updated mortality experience of an established cohort of 2,650 mine and mill workers at four 3M manufacturing facilities examined the potential association between cumulative RCS exposure and mortality from causes of *a priori* interest including: lung cancer, non-malignant respiratory disease and non-malignant renal disease. Overall, the updated study findings for these three causes of death categories were consistent with those reported in the original study [21].

No evidence of a positive exposure-response trend for lung cancer mortality by increasing cumulative RCS exposure was observed. The lack of an association with lung cancer may be explained, in part, by the relatively low exposure levels experienced in this worker cohort, as the geometric mean, 90^th^ percentile, and maximum cumulative RCS exposures, were only 0.17 mg/m^3^-years, 1.10 mg/m^3^-years, and 5.31 mg/m^3^-years, respectively. Other possible explanations for the lack of an exposure-response trend include a healthy worker survivor effect, depletion of susceptible persons after prolonged exposure, and high background rates of disease among the unexposed population [23].

Other studies have also reported a lack of an association between lung cancer mortality and cumulative RCS exposure at levels comparable to the present study [10, 19, 24]. In a pooled analysis, Steenland et al. [10] estimated the optimal threshold for lung cancer mortality was 0.33 mg/m^3^-years, which over a working lifetime would require an allowable exposure level of 0.01 mg/m^3^. However, Steenland later cautioned that the lack of an association between lung cancer and low cumulative RCS exposures may not be the result of a threshold response but rather the inability to describe a dose response at these lower levels due to small sample sizes [13]. This was later observed in a meta-analysis, where the pooled risk estimate (from 19 studies) for lung cancer mortality was elevated at 1.19 (95% CI: 1.02–1.39) in RCS exposures less than 0.83 mg/m^3^-years [9]. Risk estimates were not significantly increased in the higher exposure levels and tended to be more pronounced among silicotics than in non-silicotics.

In the previous analysis of this cohort, silicosis was listed as the underlying cause of death for three subjects and no silicosis deaths were reported during the most recent follow-up period. Information on contributing causes of death was not obtained in this study.

In the present analysis, HRs for non-malignant respiratory disease mortality were elevated for each cumulative RCS exposure category in both unlagged and lagged models; however, there was no significant increasing trend in mortality risk. Several other studies, with comparable RCS exposure levels to the present study, have evaluated non-malignant respiratory disease mortality in relation to cumulative RCS exposure in occupational cohorts [10, 20, 24–26]. Findings across these studies indicated that as cumulative RCS exposure approached 1.0 mg/m^3^- years (the highest exposure category in the present cohort was ≥0.799 mg/m^3^- years), risk estimates for non-malignant respiratory disease mortality ranged from 0.7 to 1.9. Evidence of a positive exposure-response trend for non-malignant respiratory disease mortality by increasing cumulative RCS exposure was observed in some studies [10, 25, 26] while no exposure-response trend was observed in other studies [20, 24].

It is worth noting that among the 83 deaths from non-malignant respiratory disease in the present study, 50 deaths were attributed to COPD, 12 were attributed to emphysema and four were attributed to chronic bronchitis. Given that tobacco smoking is the most common cause of these three respiratory diseases, any potential association between RCS exposure and mortality risk for these outcomes is difficult to evaluate since smoking could not be ascertained in this study. Lack of smoking information is a common limitation in retrospective mortality studies. This is of special concern for any study with respiratory endpoints, especially lung cancer and non-malignant respiratory diseases. In the present study, this information was not available and therefore could not be evaluated. Clearly this is a weakness of this study given the fact that there is this potential synergy between silica exposure and cigarette smoking as it relates to lung cancer and non-malignant respiratory disease [4, 6].

For non-malignant renal disease, the highest HR estimates, albeit imprecise, were observed in the second cumulative RCS exposure category (0.247-<0.382 mg/m^3^), with limited evidence of an increasing exposure-response trend. These findings were consistent with a recent meta-analysis which included 13 industrial cohort studies that evaluated the association between occupational RCS exposure and non-malignant renal disease [27]. This meta-analysis yielded an overall SMR = 1.52, 95% CI: 1.16–1.98; however, the authors noted that no increasing trend in mortality risk was reported in most of the studies that examined an exposure-response relationship.

The present study had the advantage of a quantitative exposure-response analysis for cumulative RCS exposure and an extended follow-up for mortality outcomes, with over 91,500 person-years of observation. Cumulative RCS exposure was determined through the creation of a detailed JEM, which was completed prior to the original mortality assessment of this cohort. Hewitt et al. [22] located, reviewed, and summarized the available RCS exposure data for the four manufacturing facilities. Their process to develop the JEM involved multiple steps, which included identification and collection of all relevant exposure data, estimation of respirable silica concentrations, and calculation of time-dependent summary exposure statistics for each manufacturing facility.

Other notable study strengths include: a low proportion of the cohort were lost to follow-up (<1%), thus minimizing a potential source of selection bias, and the underlying cause of death was determined for 99% of the cohort, with only 25 deaths from unknown causes.

## Conclusions

After seven decades of follow-up, this updated cohort mortality study of 2,650 roofing granule mine and mill workers provides no evidence of an increased trend in lung cancer in workers exposed to RCS using an internal reference group. Risk estimates for non-malignant respiratory disease were increased among workers with cumulative RCS exposure > 0.11 mg/m^3^-years; however, there was no significant evidence of a trend. Mortality risk estimates for non-malignant renal disease were elevated in the exposure-response analyses, but these estimates were highly imprecise due to the small number of deaths. Overall, the findings of this extended mortality update demonstrate concordance with a previous mortality assessment for this cohort.

## Supporting information

S1 Table Distribution of causes of death within “all non-malignant respiratory disease” and “non-malignant renal disease” categories by manufacturing facility, 1945–2015. (DOCX)

S2 Table Hazard Ratios (HRs) for selected causes of death by cumulative rcs exposure (mg/m^3^-years) for Belle Mead, 1945–2015. (DOCX)

S3 Table Hazard Ratios (HRs) for selected causes of death by cumulative rcs exposure (mg/m^3^-years) for Corona, 1945–2015. (DOCX)

S4 Table Hazard Ratios (HRs) for selected causes of death by cumulative rcs exposure (mg/m^3^-years) for Little Rock, 1945–2015. (DOCX)

S5 Table Hazard Ratios (HRs) for selected causes of death by cumulative rcs exposure (mg/m^3^-years) for Wausau, 1945–2015. (DOCX)

S6 Table Hazard Ratios (HRs) for selected causes of death by cumulative rcs exposure (mg/m^3^-years) among males only, 1945–2015. (DOCX)
