# Supplementary material for: Mortality among mine and mill workers exposed to respirable crystalline silica
Source: PLoS One. 2022 Oct 14;17(10):e0274103. doi: 10.1371/journal.pone.0274103 (PMC9565696; doi:10.1371/journal.pone.0274103)
Supplement: S1 Table — (DOCX) [file pone.0274103.s001.docx]

**S1 Table. Distribution of Causes of Death within “All Non-Malignant Respiratory Disease’” and “Non-Malignant**

**Renal Disease” Categories by Manufacturing Facility, 1945-2015**

Belle Little

ICD9 codes Total Mead Corona Rock Wausau

All Non-malignant

respiratory disease 460-519 105 11 33* 36 26*

Influenza and pneumonia 480-487 22 2 6 11 3

Bronchitis, emphysema 490-493 16 2 3 6 5

and asthma

Other non-malignant 460-466, 470-478 67 7 24* 19 18*

respiratory disease 494-496, 500-519

COPD 496 50 5 17* 13 16*

Silicosis 502 3 0 1 1 1

Aspiration pneumonia 507 3 1 0 1 1

Pleurisy 511 1 0 0 1 0

Pulmonary fibrosis 515 5 0 5 0 0

Other diseases 519 5 1 1 3 0

Non-malignant renal disease 580-589 26 1 11 12 2

Chronic glomerulonephritis 582 1 0 0 1 0

Acute renal failure 584 2 0 1 0 1

Chronic kidney disease 585 11 0 8 2 1

Renal failure NOS 586 12 1 2 9 0

*One person was common between Corona and Wausau who died of COPD

Note: NOS = not otherwise specified
