# Supplementary material for: Mortality among mine and mill workers exposed to respirable crystalline silica
Source: PLoS One. 2022 Oct 14;17(10):e0274103. doi: 10.1371/journal.pone.0274103 (PMC9565696; doi:10.1371/journal.pone.0274103)
Supplement: S2 Table — (DOCX) [file pone.0274103.s002.docx]

**S2 Table. Hazard Ratios (HRs) for Selected Causes of Death by Cumulative RCS Exposure (mg/m^3^-years) for Belle Mead, 1945-2015**

| Cumulative RCS exposure (mg/m^3^-years) | No lag | | | 15-year lag | | |
| --- | --- | --- | --- | --- | --- | --- |
|  | Deaths (n) |  | | Deaths (n) |  | |
|  |  | HR | 95% CI |  | HR | 95% CI |
| Lung cancer |  |  |  |  |  |  |
| <0.089 | 9 | 1.00 | referent | 10 | 1.00 | referent |
| 0.089-<0.224 | 6 | 1.40 | 0.49-3.98 | 7 | 1.49 | 0.56-3.98 |
| 0.224-<0.456 | 5 | 2.52 | 0.81-7.78 | 2 | 0.87 | 0.19-4.06 |
| ≥0.456 | 1 | 0.35 | 0.04-2.78 | 1 | 0.31 | 0.04-2.44 |
| p-value for trend |  | 0.31 |  |  | 0.23 |  |
|  |  |  |  |  |  |  |
| Non-malignant respiratory disease  (excluding influenza/pneumonia) |  |  |  |  |  |  |
| <0.108 | 4 | 1.00 | referent | 4 | 1.00 | referent |
| 0.108-<0.344 | 3 | 1.73 | 0.37-8.00 | 3 | 1.64 | 0.35-7.79 |
| 0.344-<0.799 | 2 | 2.22 | 0.37-13.41 | 2 | 2.59 | 0.42-16.00 |
| >0.799 | 0 | -- | -- | 0 | -- | -- |
| p-value for trend |  | --^*^ |  |  | --* |  |

^*^ Trend test was not performed due to an insufficient sample size in one or more exposure categories.

Note: Models were adjusted for sex, race, age at start of follow-up, and calendar year at start of follow-up.

Non-malignant renal disease was not analyzed at Belle Mead since there was only 1 reported death from this cause.
