# Supplementary material for: Mortality among mine and mill workers exposed to respirable crystalline silica
Source: PLoS One. 2022 Oct 14;17(10):e0274103. doi: 10.1371/journal.pone.0274103 (PMC9565696; doi:10.1371/journal.pone.0274103)
Supplement: S3 Table — (DOCX) [file pone.0274103.s003.docx]

**S3 Table. Hazard Ratios (HRs) for Selected Causes of Death by Cumulative RCS Exposure (mg/m^3^-years) for Corona, 1945-2015**

| Cumulative RCS exposure (mg/m^3^-years) | No lag | | | 15-year lag | | |
| --- | --- | --- | --- | --- | --- | --- |
|  | Deaths (n) |  | | Deaths (n) |  | |
|  |  | HR | 95% CI |  | HR | 95% CI |
| Lung cancer |  |  |  |  |  |  |
| <0.089 | 5 | 1.00 | referent | 6 | 1.00 | referent |
| 0.089-<0.224 | 8 | 1.32 | 0.37-4.81 | 4 | 0.33 | 0.09-1.26 |
| 0.224-<0.456 | 5 | 1.45 | 0.38-5.58 | 4 | 0.65 | 0.18-2.44 |
| ≥0.456 | 4 | 0.58 | 0.14-2.50 | 4 | 0.30 | 0.08-1.15 |
| p-value for trend |  | 0.21 |  |  | 0.26 |  |
|  |  |  |  |  |  |  |
| Non-malignant respiratory disease  (excluding influenza/pneumonia) |  |  |  |  |  |  |
| <0.108 | 4 | 1.00 | referent | 5 | 1.00 | referent |
| 0.108-<0.344 | 9 | 1.85 | 0.53-6.52 | 8 | 1.27 | 0.39-4.11 |
| 0.344-<0.799 | 6 | 2.23 | 0.58-8.50 | 6 | 1.69 | 0.49-5.88 |
| >0.799 | 8 | 1.87 | 0.52-6.68 | 8 | 1.45 | 0.45-4.71 |
| p-value for trend |  | 0.56 |  |  | 0.57 |  |
|  |  |  |  |  |  |  |
| Non-malignant renal disease |  |  |  |  |  |  |
| <0.247 | 2 | 1.00 | referent | 1 | 1.00 | referent |
| 0.247-<0.382 | 0 | -- | -- | 0 | -- | -- |
| 0.382-<0.714 | 3 | 6.51 | 1.07-39.64 | 4 | 17.06 | 1.89-154.30 |
| >0.714 | 6 | 4.88 | 0.97-24.45 | 5 | 7.62 | 0.88-65.88 |
| p-value for trend |  | --* |  |  | --* |  |

^*^Trend test was not performed due to an insufficient sample size in one or more exposure categories.

Note: Models were adjusted for sex, race, age at start of follow-up, and calendar year at start of follow-up.
