# Supplementary material for: Mortality among mine and mill workers exposed to respirable crystalline silica
Source: PLoS One. 2022 Oct 14;17(10):e0274103. doi: 10.1371/journal.pone.0274103 (PMC9565696; doi:10.1371/journal.pone.0274103)
Supplement: S4 Table — (DOCX) [file pone.0274103.s004.docx]

**S4 Table. Hazard Ratios (HRs) for Selected Causes of Death by Cumulative RCS Exposure (mg/m^3^-years) for Little Rock, 1945-2015**

| Cumulative RCS exposure (mg/m^3^-years) | No lag | | | 15-year lag | | |
| --- | --- | --- | --- | --- | --- | --- |
|  | Deaths (n) |  | | Deaths (n) |  | |
|  |  | HR | 95% CI |  | HR | 95% CI |
| Lung cancer |  |  |  |  |  |  |
| <0.089 | 12 | 1.00 | referent | 14 | 1.00 | referent |
| 0.089-<0.224 | 11 | 1.14 | 0.49-2.64 | 8 | 0.64 | 0.26-1.55 |
| 0.224-<0.456 | 10 | 1.12 | 0.46-2.74 | 15 | 1.10 | 0.50-2.45 |
| ≥0.456 | 18 | 1.41 | 0.63-3.14 | 9 | 0.55 | 0.22-1.35 |
| p-value for trend |  | 0.38 |  |  | 0.24 |  |
|  |  |  |  |  |  |  |
| Non-malignant respiratory disease  (excluding influenza/pneumonia) |  |  |  |  |  |  |
| <0.108 | 7 | 1.00 | referent | 8 | 1.00 | referent |
| 0.108-<0.344 | 7 | 1.01 | 0.34-3.01 | 9 | 1.04 | 0.38-2.82 |
| 0.344-<0.799 | 9 | 1.45 | 0.48-4.35 | 5 | 0.64 | 0.19-2.17 |
| >0.799 | 2 | 0.76 | 0.15-3.96 | 2 | 0.64 | 0.13-3.30 |
| p-value for trend |  | 0.88 |  |  | 0.47 |  |
|  |  |  |  |  |  |  |
| Non-malignant renal disease |  |  |  |  |  |  |
| <0.247 | 3 | 1.00 | referent | 4 | 1.00 | referent |
| 0.247-<0.382 | 7 | 10.68 | 2.27-50.22 | 7 | 7.27 | 1.75-30.25 |
| 0.382-<0.714 | 2 | 1.31 | 0.18-9.73 | 1 | 0.43 | 0.04-4.47 |
| >0.714 | 0 | -- | -- | 0 | -- | -- |
| p-value for trend |  | --* |  |  | --* |  |

^*^Trend test was not performed due to an insufficient sample size in one or more exposure categories.

Note: Models were adjusted for sex, race, age at start of follow-up, and calendar year at start of follow-up.
