# Supplementary material for: Mortality among mine and mill workers exposed to respirable crystalline silica
Source: PLoS One. 2022 Oct 14;17(10):e0274103. doi: 10.1371/journal.pone.0274103 (PMC9565696; doi:10.1371/journal.pone.0274103)
Supplement: S5 Table — (DOCX) [file pone.0274103.s005.docx]

**S5 Table. Hazard Ratios (HRs) for Selected Causes of Death by Cumulative RCS Exposure (mg/m^3^-years) for Wausau, 1945-2015**

| Cumulative RCS exposure (mg/m^3^-years) | No lag | | | 15-year lag | | |
| --- | --- | --- | --- | --- | --- | --- |
|  | Deaths (n) |  | | Deaths (n) |  | |
|  |  | HR | 95% CI |  | HR | 95% CI |
| Lung cancer |  |  |  |  |  |  |
| <0.089 | 3 | 1.00 | referent | 4 | 1.00 | referent |
| 0.089-<0.224 | 3 | 1.07 | 0.21-5.34 | 4 | 1.09 | 0.27-4.38 |
| 0.224-<0.456 | 10 | 6.22 | 1.68-22.95 | 7 | 3.56 | 1.03-12.30 |
| ≥0.456 | 6 | 1.03 | 0.25-4.23 | 6 | 0.84 | 0.23-3.06 |
| p-value for trend |  | 0.26 |  |  | 0.38 |  |
|  |  |  |  |  |  |  |
| Non-malignant respiratory disease  (excluding influenza/pneumonia) |  |  |  |  |  |  |
| <0.108 | 5 | 1.00 | referent | 6 | 1.00 | referent |
| 0.108-<0.344 | 4 | 1.02 | 0.26-3.93 | 3 | 0.63 | 0.15-2.57 |
| 0.344-<0.799 | 4 | 3.09 | 0.79-12.17 | 4 | 2.53 | 0.67-9.50 |
| >0.799 | 10 | 2.23 | 0.72-6.90 | 10 | 1.84 | 0.63-5.31 |
| p-value for trend |  | 0.13 |  |  | 0.14 |  |

Note: Models were adjusted for sex, race, age at start of follow-up, and calendar year at start of follow-up.

Non-malignant renal disease was not analyzed at Wausau since there were only 2 reported deaths from this cause.
