# Supplementary material for: Mortality among mine and mill workers exposed to respirable crystalline silica
Source: PLoS One. 2022 Oct 14;17(10):e0274103. doi: 10.1371/journal.pone.0274103 (PMC9565696; doi:10.1371/journal.pone.0274103)
Supplement: S6 Table — (DOCX) [file pone.0274103.s006.docx]

**S6 Table. Hazard Ratios (HRs) for Selected Causes of Death by Cumulative**

**RCS Exposure (mg/m^3^-years) among Males Only, 1945-2015**

| Cumulative Exposure (mg/m^3^ – years) | 1945-2015 | | |
| --- | --- | --- | --- |
|  | Deaths (n) | HR | 95% CI |
| Lung cancer |  |  |  |
| No lag |  |  |  |
| <0.089 | 26 | 1.00 | referent |
| 0.089-<0.224 | 28 | 1.19 | 0.69-2.04 |
| 0.224-<0.456 | 28 | 1.74 | 1.00-3.01 |
| >0.456 | 29 | 0.90 | 0.52-1.55 |
| p-value for trend |  | 0.33 |  |
| 15-year lag |  |  |  |
| <0.089 | 32 | 1.00 | referent |
| 0.089-<0.224 | 23 | 0.74 | 0.43-1.27 |
| 0.224-<0.456 | 27 | 1.21 | 0.71-2.05 |
| >0.456 | 20 | 0.48 | 0.27-0.85 |
| p-value for trend |  | 0.01 |  |
| Non-malignant respiratory disease (excluding influenza/pneumonia) |  |  |  |
| No lag |  |  |  |
| <0.108 | 17 | 1.00 | referent |
| 0.108-<0.344 | 22 | 1.44 | 0.76-2.73 |
| 0.344-<0.799 | 21 | 2.04 | 1.06-3.93 |
| >0.799 | 20 | 1.78 | 0.91-3.45 |
| p-value for trend |  | 0.15 |  |
| 15-year lag |  |  |  |
| <0.108 | 20 | 1.00 | referent |
| 0.108-<0.344 | 22 | 1.19 | 0.64-2.19 |
| 0.344-<0.799 | 17 | 1.38 | 0.72-2.68 |
| >0.799 | 20 | 1.51 | 0.80-2.86 |
| p-value for trend |  | 0.22 |  |
| Non-malignant renal disease |  |  |  |
| No lag |  |  |  |
| <0.247 | 5 | 1.00 | referent |
| 0.247-<0.382 | 6 | 5.97 | 1.80-19.82 |
| 0.382-<0.714 | 6 | 3.67 | 1.09-12.35 |
| >0.714 | 7 | 3.15 | 0.97-10.09 |
| p-value for trend |  | 0.13 |  |
| 15-year lag |  |  |  |
| <0.247 | 5 | 1.00 | referent |
| 0.247-<0.382 | 6 | 5.72 | 1.72-19.06 |
| 0.382-<0.714 | 6 | 3.65 | 1.09-12.23 |
| >0.714 | 6 | 2.67 | 0.80-8.89 |
| p-value for trend |  | 0.28 |  |
